# Effectiveness of a multimodal standard nursing program on health-related quality of life in Chinese mainland female patients with breast cancer: protocol for a single-blind cluster randomized controlled trial

**DOI:** 10.1186/s12885-016-2726-y

**Published:** 2016-08-31

**Authors:** Kaina Zhou, Duolao Wang, Xiaole He, Lanting Huo, Jinghua An, Minjie Li, Wen Wang, Xiaomei Li

**Affiliations:** 1 Xi’an Jiaotong University Health Science Centre, No. 76 Yanta West Road, Xi’an, Shaanxi 710061 China; 2 Liverpool School of Tropical Medicine, Pembroke Place, Liverpool, L3 5QA UK

**Keywords:** Breast cancer, Cluster randomized controlled trial, Health belief model, Health-related quality of life, Multimodal standard nursing program, Study protocol

## Abstract

**Background:**

Breast cancer and its treatment-related adverse effects are harmful to physical, psychological, and social functioning, leading to health-related quality of life (HRQoL) impairment in patients. Many programs have been used with this population for HRQoL improvement; however, few studies have considered the physical, psychological, and social health domains comprehensively, and few have constructed multimodal standard nursing interventions based on specific theories. The purpose of this trial is to examine the effect of a health belief model (HBM)-based multimodal standard nursing program (MSNP) on HRQoL in female patients with breast cancer.

**Methods:**

This is a two-arm single-blind cluster randomized controlled trial (cRCT) in clinical settings. Twelve tertiary hospitals will be randomly selected from the 24 tertiary hospitals in Xi’an, China, and allocated to the intervention arm and control arm using a computer-generated random numbers table. Inpatient female patients with breast cancer from each hospital will receive either MSNP plus routine nursing care immediately after recruitment (intervention arm), or only routine nursing care (control arm). The intervention will be conducted by trained nurses for 12 months. All recruited female patients with breast cancer, participating clinical staff, and trained data collectors from the 12 hospitals will be blind with respect to group allocation. Patients of the control arm will not be offered any information about the MSNP during the study period to prevent bias. The primary outcome is HRQoL measured through the Functional Assessment of Cancer Therapy-Breast version 4.0 at 12 months. Secondary outcomes include pain, fatigue, sleep, breast cancer-related lymphedema, and upper limb function, which are evaluated by a visual analogue scale, the circumference method, and the Constant-Murley Score.

**Discussion:**

This trial will provide important evidence on the effectiveness of multimodal nursing interventions delivered by nurses in clinical settings. Study findings will inform strategies for scaling up comprehensive standard intervention programs on health management in the population of female patients with breast cancer.

**Trial registration:**

Chictr.org.cn ChiCTR-IOR-16008253 (April 9, 2016)

## Background

Breast cancer is the most common malignant tumor in the female population. Global statistics in 2012 indicated that about 1.7 million new cases were diagnosed and 522,000 died from the disease [1, 2]. In China, as in most other countries, breast cancer is prevalent in women. According to a report in 2014, Chinese cases accounted for 12.2 % of all newly diagnosed breast cancer cases and 9.6 % of all deaths from breast cancer worldwide [3]. Over the course of illness and treatment, breast cancer patients experience many acute and chronic adverse effects. They also face unique challenges to health and well-being as a result of their cancer, its treatment, and comorbidities [4–7].

Health-related quality of life (HRQoL) is broadly conceptualized as individuals’ perceptions of their physical health, psychological health, social relationships, relationship to their environment, independence level, and personal beliefs [8]. With changing medical models, HRQoL has been regarded as a key index for evaluating global therapeutic effects and survival status in populations of patients with cancer [9]. Given negative influences of the illness and treatment, breast cancer patients experience pain, fatigue, negative psychological states, self-image alteration, body function limitations, self-esteem reduction, and risk of recurrence, which severely impact physical, psychological, and social functioning [10–17]. Breast cancer patients also have been shown to have poorer HRQoL in comparison with the general population, especially among patients under 50 years of age [18, 19].

To improve HRQoL for breast cancer patients, many programs have been used with this population, such as art therapy (e.g., music therapy, dance/movement therapy) [20–22], exercise interventions (e.g., physical exercise/activity, resistance exercise, aerobic exercise, yoga) [23–28], psychoeducational support (e.g., health education, psychosocial support, spiritual group therapy) [29–33], and multimodal programs (e.g., rehabilitation programs, physiotherapy programs, exercise programs) [34–37], with different effects on HRQoL. However, existing or potential health problems in physical, psychological, and social domains have not been considered comprehensively. Additionally, the previously mentioned programs fail to describe intervention parameters such as time, frequency, or strength in an explicit manner, leading to their unsuitability as standard nursing interventions. Moreover, few studies have constructed a multimodal standard nursing program (MSNP) for breast cancer patient populations [38].

### Theoretical framework

The MSNP is developed based on the Health Belief Model (HBM), which attempts to explain and predict health behaviors by focusing on the attitudes and beliefs of individuals. The HBM comprises four constructs: perceived susceptibility, perceived severity, perceived benefits, and perceived barriers. These concepts are proposed to account for people’s readiness to act [39]. Cues to action are thought to activate readiness and stimulate overt behavior. Self-efficacy or confidence promotes the performance of an action. Moreover, a person will take a health-related action if it is felt that a negative health condition can be avoided, there is a positive expectation about taking a recommended action, and a belief exists that a recommended health action can be taken successfully [40].

**Fig. 1:**
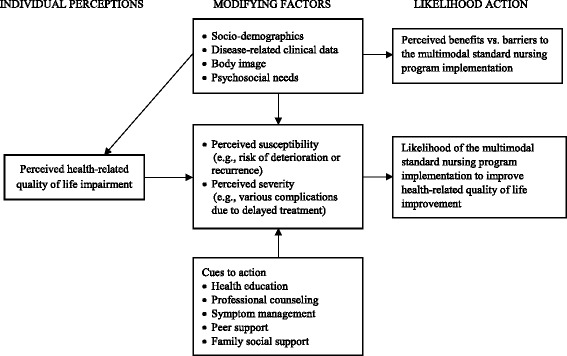
Conceptual framework for the multimodal standard nursing program (MSNP) development

The HBM has been widely used in different populations [41–45]. However, it has rarely been employed as a theoretical guide in developing nursing intervention strategies in patients with breast cancer [46–48]. Based on the HBM, influencing factors can be explored from physical, psychological, and social viewpoints. Therefore, perceived susceptibility (e.g., risk of deterioration or recurrence), perceived severity (e.g., complications due to delayed treatment), perceived benefits (e.g., positive feedback following MSNP implementation), and perceived barriers (e.g., impediments to MSNP implementation) can be identified during the treatment and nursing process to aid in the development of the MSNP (Fig. 1).

### Aims and objectives

The aim of this trial is to evaluate the effectiveness of a MSNP for female patients with breast cancer in Xi’an, China. The primary objective is to test for effects of the MSNP on HRQoL enhancement. The secondary objectives are to assess improvements in pain, fatigue, sleep, breast cancer-related lymphedema (BCRL), and upper limb function.

Based on these objectives, the primary hypothesis is that breast cancer patients receiving MSNP will achieve better HRQoL than a control arm at 12 months. The secondary hypotheses are that patients receiving the intervention will have (i) improved HRQoL at 1, 3, and 6 months; (ii) lower pain scores and less BCRL occurrence; and (iii) improved fatigue, sleep, and upper limb function at 1, 3, 6, and 12 months.

## Methods

### Design

This is a two-arm single-blind cluster randomized controlled trial (cRCT) in clinical settings with female breast cancer patients.

### Participants

Participants are inpatients with breast cancer. Inclusion criteria are newly diagnosed with breast cancer, female, aged 18 years and older, preparing to receive radical mastectomy and other auxiliary treatments (e.g., chemotherapy, radiotherapy, endocrine therapy), and providing written informed consent. Patients with cognitive disorders, psychiatric disorders, other malignant tumors, active infection, or other severe potential infection will be excluded. Cognitive disorders and psychiatric disorders will be screened using DSM-V (Diagnostic and Statistical Manual of Mental Disorders, 5^th^ ed.) criteria [49]. Reasons for refusal to participate will be documented.

### Sample size and randomization

The sample size was calculated based on the FACT-Bv4.0 total score of similar intervention studies (two-arm trials with Chinese mainland female patients with breast cancer employing a 12-month follow-up period). According to an eligible study [50], 74 patients (37 in each arm) will be needed to detect a between-arm change of 6.74 in the FACT-Bv4.0 total score with a power of 80 % at a 5 % level of statistical significance. The sample size was increased to 90 patients (45 in each arm) to allow for a 20 % dropout rate. Assuming an intra-cluster correlation coefficient (ICC) of about 0.1 and 30 patients per cluster, the sample size adjusting for clustering is 12 clusters or 360 patients [51]. That is, a total of 12 tertiary hospitals will be required in the trial, with 6 hospitals in each arm and 30 patients per hospital on average.

The 12 tertiary hospitals will be randomly selected from the 24 tertiary hospitals in Xi’an district and allocated to the intervention and control arms using a computer-generated random numbers table. Selection and allocation of the 12 hospitals will be carried out independently by a member of the research team.

### Procedure

**Fig. 2:**
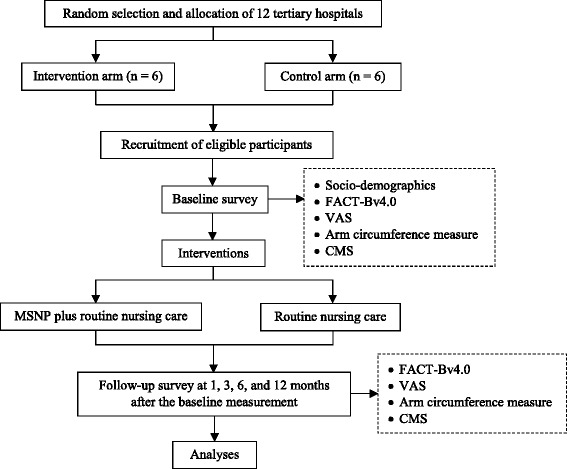
Flowchart of the trial. (FACT-Bv4.0: Functional Assessment of Cancer Therapy-Breast version 4.0; VAS: visual analogue scale; CMS: Constant-Murley Score; MSNP: multimodal standard nursing program.)

Following random selection and allocation of the 12 tertiary hospitals, eligible female inpatients with breast cancer from each hospital will receive either MSNP plus routine nursing care immediately after recruitment (intervention arm), or only routine nursing care (control arm). The patients in both arms will provide demographic data and complete the pre-test of the FACT-Bv4.0, VAS, arm circumference, and CMS prior to the intervention. After the baseline measurement, four post-tests (i.e., FACT-Bv4.0, VAS, arm circumference, and CMS) at 1, 3, 6, and 12 months will be conducted. Items on the questionnaire will be asked of all patients and their answers will be recorded by trained data collectors. The flow chart of the cRCT procedure is depicted in Fig. 2.

### Outcomes and measurements

The primary outcome is HRQoL measured by the Functional Assessment of Cancer Therapy-Breast version 4.0 (FACT-Bv4.0) at 12 months.

#### FACT-Bv4.0

The 36-item Chinese (simple) FACT-Bv4.0 consists of a general cancer subscale (FACT-G) and a breast-cancer-specific subscale for additional concerns (BCS). The FACT-G comprises physical well-being (PWB, seven items), social/family well-being (SFWB, seven items), emotional well-being (EWB, six items), and functional well-being (FWB, seven items). The BCS comprises nine items, each rated on a 5-point Likert scale (from 0 to 4). The FACT-Bv4.0 total score, which is the sum of scores of the FACT-G and BCS ranges from 0 to 144. A higher score indicates better HRQoL of the patient [52]. The reliability, validity, and responsiveness of the Chinese (simple) FACT-Bv4.0 has been confirmed in Chinese patients with breast cancer [53].

The secondary outcomes include pain, fatigue, sleep, BCRL, and upper limb function evaluated by a visual analogue scale (VAS), the circumference method, and the Constant-Murley Score (CMS) at 1, 3, 6, and 12 months.

#### VAS

The VAS is a psychometric response scale used to measure subjective characteristics or attitudes that cannot be directly assessed. When answering a VAS item, respondents specify their level of agreement with a statement by indicating a position along a continuous line between two end-points [54]. In this study, a 0–10 cm VAS will be used in the subjective evaluation of pain (no pain to severe pain), fatigue (no fatigue to severe fatigue) and sleep (good sleep to poor sleep) in the patients.

#### Circumference method

Arm circumference will be measured at 10 cm above the wrist and 10 cm above the elbow using a leather measuring tape. Lymphedema is defined as a difference in arm circumference of more than 2 cm between the treated and untreated side at either of the two measured locations on the limb [55]. The measurement of arm circumference of all recruited patients will be consistently performed at 5 pm at the baseline survey and at each post-test.

#### CMS

The Chinese CMS has four subscales, including pain (15 points maximum), activities of daily living (20 points maximum), range of motion (ROM, 40 points maximum), and strength (25 points maximum). The total score ranges from 0 to 100, with a higher score indicating a higher quality of functioning [56]. A systematic review showed that the original CMS satisfied psychometric properties of functional assessment [57].

### Interventions

#### The intervention arm

Patients in the intervention arm will receive the MSNP based on routine nursing care delivered by trained clinical nurses immediately after recruitment. The MSNP includes physical care, help with psychological adjustment, and social support, aiming to improve the physical, psychological, and social functioning of the patients, respectively. Detailed information on the MSNP content and implementation are shown in Table 1.

**Table 1:** Outline of the multimodal standard nursing program (MSNP)

Section	Target	Content	Implementation	Special attention
Physical care	Physical function	Systematic functional exercises		
		• Upper limb exercise (two-sides)	Exercises of the finger, wrist, forearm, elbow, upper arm, shoulder, head & neck; 5–10 times per day, 15 min per session.	Patients with complications and abnormal conditions should limit the time and strength of exercise. Continue to the end of follow-up.
		• Aerobic exercise	Walking up and down stairs, 3–6 times per day, 30 min per session.	
		• Progressive muscle relaxation	Sitting or lying, relaxing from head to feet, 3–6 times per day, 30 min per session.	
Psychological adjustment	Psychological function	Psychological counseling	Nurse-conducted one-to-one communication on the patient’s psychological problems; twice per week, 30–60 min per session in the hospital. Need-oriented counseling is delivered via outpatient review or telephone after discharge from the hospital.	Continue to the end of follow-up.
		Music listening	Listening to patient’s preferred light music via MP3 player; twice per day (7 a.m.–9 a.m. and 9 p.m.–11 p.m.), 30 min per session.	Patients with sound allergy or who dislike listening to music should not be given such intervention. Continue to the end of follow-up.
		Interactive distraction	Need-oriented communication with caregivers or peers while in a negative mood.	Continue to the end of follow-up.
Social support	Social family function	Family support training	Training caregivers on the monitoring of the patient’s diet, exercise, rest and illness, as well as coping with the negative influences of breast cancer on families. Once per week, 60 min per session in the hospital. Need-oriented training is delivered via outpatient review after discharge from the hospital.	Continue to the end of follow-up.
		Peer group support	Rehabilitation experiences exchange between the patient and peers, discussing successful recovery in a chatty manner. Once per week, 60 min per session in hospital setting. Need-oriented support is delivered via outpatient review after discharge from the hospital.	Continue to the end of follow-up.

#### The control arm

Patients in the control arm will only receive routine nursing care, including vital signs observation, nursing specific to surgery, drainage tube nursing, fundamental exercises after surgery, and post-operative complications monitoring.

### Masking

All recruited female patients with breast cancer, participating clinical staff, and trained data collectors of the 12 hospitals will be blinded to the allocation information. Patients in the control arm will not be offered any information on the MSNP during the study period in case of bias.

The trial statistician will also be blinded to the treatment code during development of the statistical analysis plan and writing of the statistical programs, which will be validated and completed using dummy randomization codes. The actual allocation will only be provided to the study team after locking of the database.

### Data management and analyses

SAS 9.4 and SPSS 22.0 will be employed to perform all statistical analyses. All quantitative data will be collected using paper questionnaires with unique ID numbers for each recruited patient. Data will be stored at the research team office at the end of each day. Daily checking of data will be carried out by the research coordinator, with queries identified and resolved promptly. A database will be built using Epidata3.1; double entry and checking will be performed by an assigned data entry team. Discrepancies will be resolved by a third data manager. Once in an electronic file, all data will be password protected, with data managers controlling access to passwords and ensuring the database is backed up daily.

Findings of the trial will be reported according to the CONSORT guidelines for cRCT. Primary analyses will be based on an intention-to-treat (ITT) population and secondary analyses on a per-protocol (PP) population. The primary endpoint will be analyzed using a linear mixed model with intervention, time, and interaction between intervention and time as fixed effects, baseline measurement as covariate, and cluster and patient as random effects. The intervention difference at each time, together with its 95 % confidence interval will be derived from the mixed model. Missing data will be treated as missing at random in the above mixed model analysis and no imputation will be made. To assess the sensitivity of the result of this assumption, the last observation carried forward (LOCF) strategy will be used to compute missing HRQoL scores during follow-up. A covariate-adjusted mixed model of the primary endpoint will be tested by adding pre-specified covariates at baseline into the linear mixed model. Subgroup analysis will be performed on the pre-specified covariates.

Continuous secondary outcomes will be analyzed in a similar way to the primary endpoint analysis. For the analysis of binary secondary outcomes, a generalized mixed model will be employed with intervention, time, and the interaction between intervention and time as fixed effects, baseline measurement as covariate, and cluster and patient as random effects. The odds ratio (OR) between the two arms at each time, together with its 95 % CI will be derived from the generalized mixed model.

All analyses will be described in detail in the finalized and signed statistical analysis plan before unmasking the study.

### Ethical approval

The trial protocol received ethical approval from the Biomedical Ethics Committee of Xi’an Jiaotong University Health Science Center. Written informed consent will be obtained from each recruited patient before the intervention and questionnaire survey.

## Discussion

To improve HRQoL in female patients with breast cancer, novel intervention strategies that can improve physical, psychological, and social functions as well as sustain those improvements over time are required. This trial will examine the effectiveness of an MSNP in improving HRQoL compared to routine nursing care. The efficacy of the intervention on pain, fatigue, sleep, BCRL, and upper limb function will also be tested. The MSNP has several advantages over routine nursing care: (i) it is constructed based on validated evidence and within the theoretical framework of the HBM; (ii) taking into consideration physical, psychological, and social health domains, it outlines comprehensive nursing strategies for HRQoL improvement mainly in clinical contexts; and (iii) it may improve self-management of health in female patients with breast cancer, because patients will receive follow-up health instructions from nurses at home or in community settings.

Even in the case of null results, this trial will produce a large amount of illuminating data. Investigators will be able to closely monitor HRQoL in both arms during the 12-month follow-up period. If the MSNP is effective, it will provide an additional nursing care option for comprehensive health management of the population of female patients with breast cancer.

## Abbreviations

BCRL: Breast cancer-related lymphedema
CMS: Constant-Murley Score
cRCT: Cluster randomized controlled trial
FACT-Bv4.0: Functional Assessment of Cancer Therapy-Breast version 4.0
HBM: Health Belief Model
HRQoL: Health-related quality of life
MSNP: Multimodal standard nursing program
VAS: Visual analogue scale
